# Putative role of conserved water molecules in the hydration and inter-domain recognition of mono nuclear copper centers in O2-bound human ceruloplasmin: A comparative study between X-ray and MD simulated structures

**DOI:** 10.6026/97320630015402

**Published:** 2019-06-15

**Authors:** Bishnu P. Mukhopadhyay

**Affiliations:** 1 Department of Chemistry National Institute of Technology-Durgapur, West Bengal, Durgapur - 713209, India

**Keywords:** Ceruloplasmin, multicopper oxidase, Type-1 copper centers, MD- simulation, conserved water molecules

## Abstract

Human Ceruloplasmin (hCP) is an unique multicopper oxidase which involves in different biological functions e.g., iron metabolism, copper transportation, biogenic amine oxidation ,and its malfunction causes Wilson's and Menkes diseases. MD- simulation studies of O2- bound solvated structure have revealed the role of several conserved/ semi-conserved water molecules in the hydration of type-I copper centers and their involvement to recognition dynamics of these metal centers. In O2- bound structure, hydration potentiality of CuRS (Cu1106) type-I copper center is observed to be unique, where two water molecules (W1-W2) are interacting with the metal sites, which was not found in X-ray structures of hCP. Generally, in the interdomain recognition of Cu_Cys-His_ to CuRS, CuRS to CuPR and CuPR to Cu_Cys-His_ centers, the copper bound His-residue of one domain interacts with the Glu-residue of other complementary domain through conserved/ semi-conserved (W3 to W5) water- mediated hydrogen bonds (Cu-His...W...Glu), however direct salt-bridge (Cu-His...Glu) interaction were observed in the X- ray structures. The MD- simulated and X- ray structures have indicated some possibilities on the Cu-His...W...Glu ↔ Cu-His...Glu transition during the interdomain recognition of type-I copper centers, which may have some importance in biology and chemistry of ceruloplasmin.

## Background

Ceruloplasmin (CP) is an unique multicopper oxidase and it is involved with iron metabolism (ferroxidase activity), copper transportation, catalyzes Cu(I) oxidation, promotes the oxidation of biogenic amines (norepinephrine, epinephrine, and serotonin) and can act as effective anti-oxidant 1. Beside these multifunctional activities of that plasma protein, it is also associated with two hereditary disorders involving copper transport: Menkes' 'kinky hair' disease and Wilson disease (hepatolenticular degeneration) 1. Till now few crystallographic structures (PDB Id: 4ENZ, 2J5W and 1KCW) of ceruloplasmin (having resolution from 2.6 to 3.2 Å) have provided some information on the coordination behaviour of residues to three mononuclear copper centers (MNC) and trinuclear copper cluster (TNC), and also focus the stabilization mechanism of six domains in that protein structure 2-4. Unlike the other mononuclear copper proteins having one T-1 Cu-center e.g., azurin, plastocyanin, rusticynin etc. which involves to electron transfer reaction, ceruloplasmin is distinctly different 5-8. Here the three T-1 copper centers (Cu _Cys-His_, Cu _RS_ (remote site) and Cu _PR_ (permanently reduced)) belonging to domains 6, 4 and 2 are separated by ~18 Å 4, 9, 10. The Cu_Cys-His_ center is ~13 Å away from the nearest copper ion of the trinuclear cluster. In hCP, the substrate molecule is thought to interact with T-1 copper site, which accepts the electrons from that molecule and transfers the electrons to trinuclear copper cluster which serves as a catalytic site to reduce O2- molecule to water, however, the detail catalytic mechanism is still unknown^2^, ^11^. The type-1 copper center in domain 4 and 6 are typical blue copper sites where each of the copper ions is coordinated by a Cys, two His residues and weakly interacts with a Met residue. In domain 2, the T-1 copper is coordinated by a Cys, two His and interacts weakly with a Leu residue. The arrangement of TNC and mononuclear copper ion in domain 6 is almost the same as were found in Ascorbate oxidase, Bilirubin oxidase and in some members of the Laccase family; though each of the structure contains only one T-1 copper center 12-14. The water molecules play an important role in the structure function activity of proteins 15-17. Specially the interaction of conserved/semiconserved water molecules to metal centers, their involvement to redox process, metal-metal or interdomain recognition have indicated the importance of water molecules in biochemical function of metalloenzymes 18. But in the O2-bound crystal structures of CP, very few numbers of water molecules are observed in the asymmetric unit, even no water molecules were observed at ~4-5 Å around the three mononuclear copper centers. Even the role of water molecules in the interdomain recognition of three Type1 copper centers was not being reflected in the X-ray studies. Previous MD-simulation study of 2J5W PDB structure of CP was indicated the hydration potentiality of trinuclear copper cluster 19, however the results could not provide any evidence on the aquation potentiality of Type1 copper centers or on the role of water molecules in the interdomain recognition of Type1 copper center which was thought to be an important aspect concerning to the biology of CP. To investigate those aspects, MD-simulation study has been followed and carried out the solvated structure of hCP (PDB Id: 4ENZ).

MD-simulation study of O2- bound solvated hCP structures can shed some light on the hydration potentiality of three T-1 copper centers and coordination behavior of water molecules to those copper centers. The investigation may also enlighten the interdomain recognition dynamics of T-1 copper bound histidine of one domain to glutamic acid residues of other domain and the role of conserved water molecules in that recognition process. All these results provide some new light on the chemistry of T-1 copper centers in CP which may have some implication to the structural biology of that multifunctional protein.

## Methodology

The X-ray crystallographic structure PDB Id: 4ENZ having 2.6 Å resolution (R = 0.20) was used for MD- simulation studies. In the asymmetric unit of crystal structure, one ceruloplasmin molecule (CP) was present along with few ions, some other small organic molecules and 86 number of water molecules. The numbering scheme for six copper ions and amino acid residues were followed in according to 4ENZ crystal structure.

## Structure preparation:

Two N-acetyl-D-glucosamine (NAG) molecules, one oxygen atom near to Cu1104, glycerol (GOL) molecules, Ca2+and Na+ ions were initially removed from the PDB structure. Then, total of 17 number of missing residues at the different sequences 476-482 (Tyr-Asn- Pro-Gln-Ser-Arg-Ser), 886-889 (Leu-Lys-Val-Phe) and 1041-1046 (Glu-Asp-Thr-Lys-Ser-Gly) were added at the proper positions of protein. Before energy minimization, the six histidine residues (coordinated to three mononuclear T-1 copper centers) were converted to HSE form, and the histidines associated to trinuclear copper cluster were assigned as HSD form. All copper atoms of the trinuclear cluster (Cu1103, Cu1105, and Cu1104) and T-1 mononuclear centers (Cu1102, Cu1106, Cu1107) in the protein structure were kept fixed. Then successive energy minimizations (of all the residues) were followed stepwise by steepest descent (1000 steps) and conjugate gradient (2000 steps) methods. The final structure of the protein was checked by superimposing it on the crystal structure, and the stereochemical arrangements of the newly added residues were verified by the Ramachandran plot.

## Quantum chemical calculation:

At first, the three truncated models (No.1-3) were prepared from the 4ENZ crystal structure by taking each of the T-1 copper atoms along with their coordinating residues like His, Cys, Met or Leu. Then the other model was built by taking all the three copper atoms of trinuclear copper (T2/T3) cluster along with their coordinating residues and O2 molecule (Model No. 4). The QM calculations were performed using B3LYP functional method with standard split valence basis set 6-31G(d) for hydrogen, carbon, nitrogen, oxygen, and sulfur atoms and valence double zeta basis set with effective core potential (LanL2DZ_ecp) 20, 21 on the copper atoms.

Solvation energies were added during optimization calculations using the conductor-like Polarizable Continuum Models (C-PCM) 22. In each model system, the copper coordinating residues were surrounded by a polarizable dielectric continuum. The standard value of dielectric constant (ε=78.39) was chosen to model the protein surrounding with water molecules. During geometry optimization, the methylated atoms for the truncated residues were kept fixed. Finally, Natural Bond Orbital (NBO) analysis 23 was done on the optimized structures to obtain the natural charges on copper atoms, and oxygen molecule, which was retained during classical MD- simulation methods. The charges of T-1 copper centers were found as: Cu1102: 0.7936, Cu1106: 0.7109, Cu1107: 1.0426 and for the trinuclear copper cluster Cu1103: 1.4303, Cu1105: 1.1613 and for Cu1104: 1.0862. The charges for two oxygen atoms in the O2 molecule O1: -0.5083 and O2: -0.5308. All the quantum chemical calculations were performed by TeraChem 24 computational program.

## Identification of conserved water molecules

The 3DSS server 25 and Swiss PDB viewer program 26 were used to locate the conserved water molecules around the mononuclear copper centers and their associated residues of the Xray and MD- simulated structures. The 4ENZ PDB-structure was taken as reference and the other MD- simulated structures were successively superimposed on it. After superposition of the simulated structures, the water molecules which were within 1.8 Å and formed at least one hydrogen bond with the protein or coordinated to T-1 copper centers were considered to be conserved or semi-conserved 27. But at certain instances, water molecules were considered to be equivalent where a similar type of hydrogen bonding pattern was encountered even if the pair-wise distance criterion was not satisfied due to varying side-chain conformation 28.

## Molecular dynamics (MD) simulation

**Figure 1:**
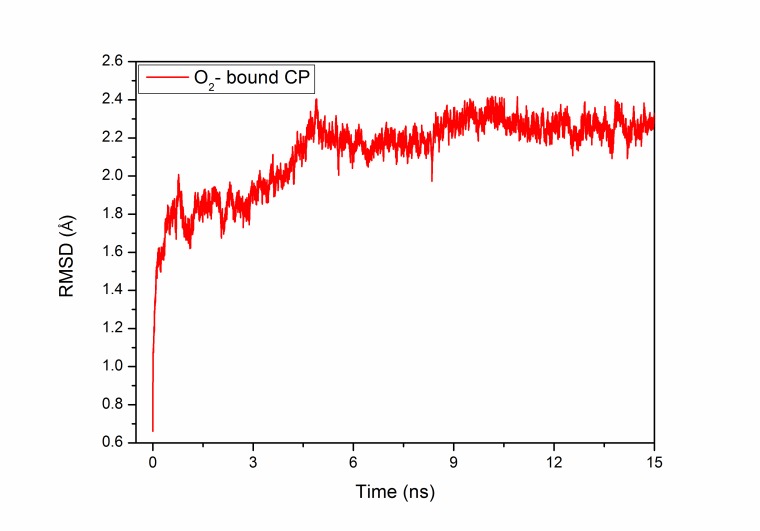
The root mean square deviation (RMSD) of the O2- bound structure of human ceruloplasmin during MD simulation.

Molecular dynamics simulations of all the structures were performed using NAMD v.2.6 29 with CHARMM36 force field 30-31. The necessary charges for six copper atoms (Cu1102 � Cu1107) and oxygen molecule were obtained from the NBO calculations (performed on the truncated optimized structures) and they were assigned at the respective copper atoms of the enzyme. All the Na+ and Ca+2 ions present in the crystal were also added in the structure before simulation. Then each structure was converted to Protein Structure File (PSF) by Automatic PSF Generation Plugin within VMD program v. 1.9.3 32. Then keeping the crystal water molecules intact, the hCP structure was solvated (with 21057 number of water molecules) using the VMD program. All the water molecules were converted to the TIP3P water model 33. Subsequent energy minimization was performed by the conjugate gradient method. Initial energy minimization was performed for 1000 steps by fixing the six copper ions, oxygen molecule and protein backbone atoms, followed by a final minimization for 2000 steps for all atoms of the system to remove the residual steric clashes. Then the structure was simulated at temperature (310 K) and pressure (1 atm) by Langevin dynamics 34 using periodic boundary condition. The Particle Mesh Ewald method was applied for full-electrostatics and the Nose-Hoover Langevin piston method was used to control the pressure and dynamical properties of the barostat 35. Then water dynamics was performed for 2 ns by fixing the copper ions and protein residues, allowing the water molecules to move freely. Finally, all atom molecular dynamics simulation for 15 ns was carried out for oxygen-bound form of hydrated hCP (4ENZ structure). The atomic coordinates of MD structures were recorded at every 1 ps interval. The residue�water and residue�residue interaction energies were calculated using the NAMD Energy Plugin in VMD. The root mean square deviation (RMSD) of MD structure was calculated (X-ray structure was taken as a reference molecule) by RMSD trajectory tool in VMD (Figure 1). The simulation trajectory was analyzed from 1 to 15 ns to locate and investigate the interaction of conserved water molecules.

## Results and Discussion:

During the simulation of O2-bound solvated structures of CP, few water molecules are observed to interact with the CuRS T-1 copper center with high residential frequencies (R.F.) which were not found in the 2J5W and 4ENZ crystal structures. Moreover in the crystal and all simulated structures, recognition of three T-1 mononuclear copper bound histidine residues (of first coordination sphere) belonging to even numbered (2,4 and 6) domains are made either through direct salt-bridge (Cu-His...Glu) or water-mediated hydrogen bonds with glutamic acid residues of other even numbered complimentary domain. However, water mediated saltbridge recognition between the histidine and glutamic acid residues (Cu-His...W...Glu) were not found in any X-ray structure. In the entire text, numbering scheme for three T-1 mononuclear copper centers (Cucis-His (Cu1107), CuRS (Cu1106) and CuPR (Cu1102)) and the residues of ceruloplasmin is followed according to 4ENZ PDB- structure. For convenience, numbering scheme of the equivalent water centers (interact with same type-1 copper bound His residue or involve in the same or similar interdomain recognition) in O2-bound CP structure are kept the same.

## Hydration susceptibility of T-1 copper centers:

**Figure 2:**
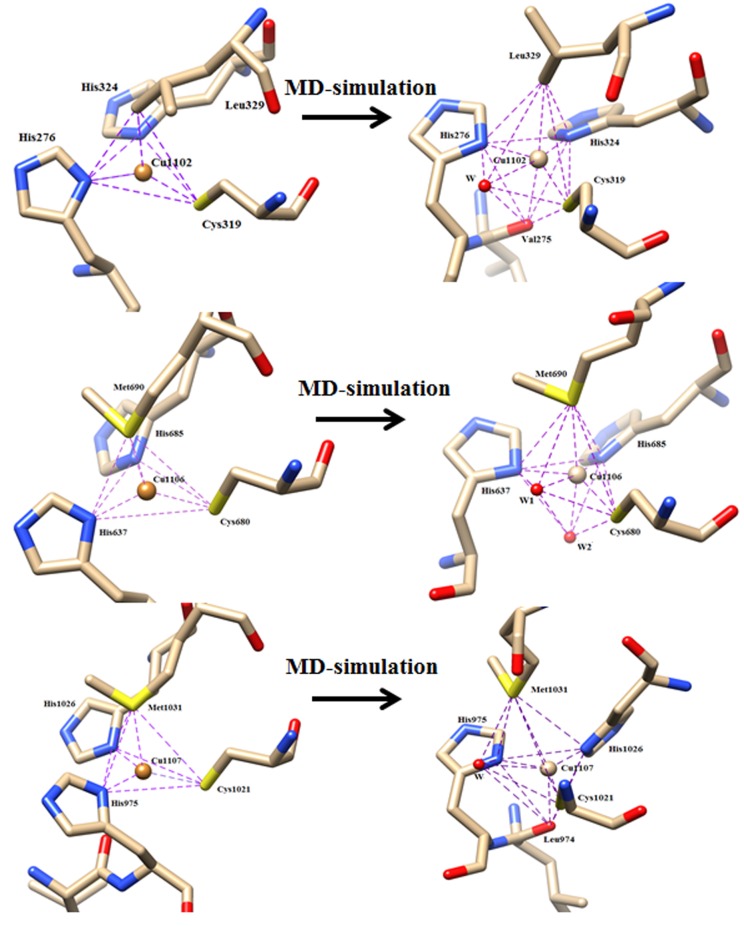
The coordination of residues at the CuRS(Cu1106) mononuclear copper center in the X-ray structure of human ceruloplasmin (left). The interaction of residues and water molecules (W1- W2) with the copper center (CuRS) after MD- simulation (right) is shown.

During the simulation of O2-bound CP-structure, two water molecules are observed to interact with CuRS (Cu1106) T-1 copper center. During simulation of the structure , it is interesting to note that one water molecule (W7769) is observed at 3.20 to 4.04Åaway (trans to His324) from the CuPR (Cu1102) center with ~60% occupation frequency (O.F.) and another water molecule (W8205) is observed at 3.48 to 4.33 Å away (trans to His1026) from the Cu_Cys-His_ (Cu1107) center (Figure 2) with reasonable occupation frequency (~60%) , however they were not observed in the crystal structures of ceruloplasmin (PDB Id: 2J5W and 4ENZ). Again, beside the coordination of His637 (ND), His685 (ND), Cys680 (SG), Met690 (SG) residues to Cu1106 (or CuRS) site (as were observed in the crystal), two water molecules W5605 and W6525 interact with that copper center and occupied the respective W1 and W2 conserved hydrophilic centers with 95 and 100% occupation frequencies. The W1 and W2 water centers interact with CuRS center from the trans direction of His685 and Met690. During the simulation, W1...CuRS and W2 ... CuRS distances are varying from 2.70 to 3.60 and 2.33 to 2.65 Å in the O2-bound structure. The Id No. of water molecules which have occupied the conserved /semi-conserved water centers (W1 and W2) and their distances from the respective copper centers are given in Table 1. This type of interaction of water molecules with T1-copper center was also noticed in the energy minimized Xray structure of a bacterial protein rusticyanin 36. In crystal or at the initial stage of simulation, coordination geometry of the residues around all the three mononuclear copper centers are observed to be distorted tetrahedral which are usually found in azurin, rusticyanin etc. 37. However, after interaction of water (W1 and W2) molecules, coordination geometry around the CuRS(Cu1106) mononuclear copper center has also been changed which was shown in Figure 2.

All these results indicate the hydration potentiality of T-1 copper center Cu1106 (or CuRS) and it seems to have higher hydrophilic susceptibility compare to other two mononuclear copper centers (CuPR (Cu1102) and CuCys-His (Cu1107)) in ceruloplasmin. The crystallographic studies of few substrate bound CP complexes have also indicated the importance of that CuRS center, where it was thought to act as an initial electron acceptor from substrate molecule 9, 38. However, our results have also provided some aspects on the hydrophilic susceptibility of that mononuclear copper center which might have some importance in the stabilization and biological function of CP.

## Recognition of three T-1 copper centers:

**Figure 3:**
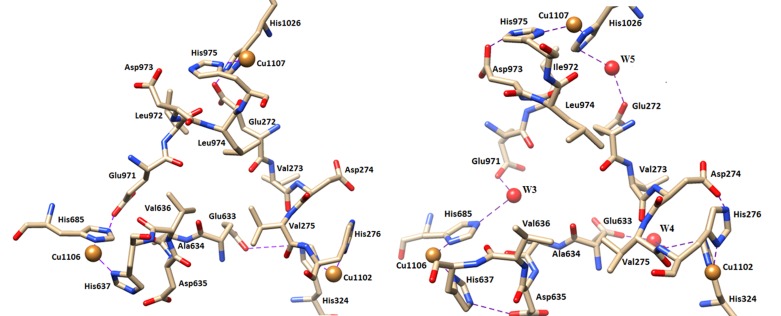
Interdomain recognition of three type I copper bound His residues in the (a) X-ray structure (left). (b) O2- bound simulated structure of ceruloplasmin (right). All the H- bonds with water (W) molecules are shown by dotted lines.

Ceruloplasmin structure is comprising with six domains (D), sequence numbers of residues in the respective domains are following: 1-192 (D1) , 193-340 (D2) , 347-553 (D3) , 554-703 (D4) ,704-884 (D5) and 891-1040 (D6)[4]. The three T-1 copper centers CuCis-His (Cu1107), CuRS (1106) , CuPR (1102) are present in the respective even numbered domains D6, D4, and D2. Generally, during the simulation the interdomain recognition of T-1 copper centers are either followed through salt-bridge or water mediated hydrogen bonding interaction between the Cu-bound His of one domain with the Glu residue of other complimentary domain. The id no. of the interacting water molecules which have occupied the conserved / semi conserved water centers (W3 to W5) are given in Table 1. The salt-bridge and other water mediated hydrogen bonding interaction of these copper bound basic His- residue of one domain to acidic Glu residue of other domain and their distances in the simulated and X-ray structures are given in Table 2. The interdomain recognition of three type I copper bound His residues in the X-ray and O2-bound simulated structures are shown in Figure 3 (a, b).

## CuCis-His (Cu1107) to CuRS (Cu1106) recognition (D6���D4 ):

In the crystal, recognition of CuCis-His (Cu1107) to CuRS (Cu1106) has been made through the direct salt-bridge- interaction between Glu971(OE1) of domain 6 with CuRS bound His685 (NE) residue of domain 4: CuCis-His-His975-Leu974-Asp973-Ile972-Glu971 ...His685 (NE)-CuRS, where the Glu971(OE2)...His685 (NE) distance was 2.80 and 3.36Å in the respective 2J5W and 4ENZ PDB-structures. During MD-simulation of O2-bound solvated structures of CP, both the OE1/OE2 atoms of Glu971 are stabilized by 2-3 water molecules through hydrogen bonds. In O2-bound structure , in most of the time, inter domain recognition between CuCis-His (Cu1107) to CuRS (Cu1106) seems to be made through the interaction between Glu971(OE1) of domain 6 with His685 (NE) of domain 4 through a semi-conserved water molecule (W3) having residential frequency ~60% : CuCis-His-His975-Leu974-Asp973-Ile972-Glu971...W3... His685(NE)-CuRS, which was not found in 2J5W or 4ENZ PDBstructures. The Glu971(OE1/OE2)...W3 and W3...His685 (NE) distances are ranging from 2.64 to 3.01 and 2.75 to 3.40 Å. However, in the interval of 6.71 to 8.80 ns, the distance between Glu971(OE1) to His685(NE) seems to be ~ 10 Å when three water molecules have connected the above two residues through hydrogen bonds. The average energy ranges of Glu971(OE2)...His685(NE2), Glu971(OE1)...W3 and W3...His685(NE2) interaction are -0.137 to - 0.155, -0.121 to -0.152 and -0.126 to -0.152 kcal/mol, respectively.

## CuRS (Cu1106) to CuPR (Cu1102) recognition (D4�.D2):

In the crystal, recognition between CuRS (Cu1106) to His324 of CuPR (Cu1102) is observed to mediate through the interaction of Glu633 (OE1) of domain 4 to His324 (NE) of domain 2 (Cu_RS_-His637- Val636-Asp635-Ala634-Glu633...His324-Cu_PR_). The Glu633(OE1)...His324(NE) distance is observed to be slightly high 4.11 and 3.58Å in the respective O2-bound 4ENZ and 2J5W PDBstructures. In the O2-bound structure, from 0-11.6 ns the Glu633 interacts similarly with His324 through one conserved water molecule (W4) and the Glu633(OE1/OE2)...W4, W4...His324(NE) distances are varied from 2.50 to 2.95 and 3.30 to 3.65Å, but from 11.63 to 15ns the Glu633 seems to be far away (~8Å) from His324 then both the residues are observed to connect by two water molecules (W4 and W4') , where the His324(NE)...W4, W4...W4' and W4'...Glu(OE2) distances are varied from 2.98 to 3.75 , 2.7 to 3.10 and 2.54 to 2.8 Å . So, in the simulated structures recognition between CuRS to CuPR may mediate either through (a) or (b):

(a) Cu_RS_-His637-Val636-Asp635-Ala634-Glu633...W4...His324(NE)- CuPR

(b) CuRS-His637-Val636-Asp635-Ala634-Glu633...W4'...W4...His324-CuPR

All these interaction and distances of water molecules from the Glu633 and His324 residues during simulation are given in Table 2. The average energy ranges of Glu633(OE1)...His324(NE2), Glu633(OE1)...W4 and W4...His324(NE2) interaction are -0.103 to - 0.114, -0.111 to -0.139 and -0.133 to -0.175 kcal/mol respectively.

## CuCis-His (Cu1107) to CuPR (Cu1102) recognition (D6���D2 ) :

In the crystal, recognition of CuCis-His (Cu1107) to CuPR (Cu1102) is made through salt-bridge interaction between the Cu1107 bound His1026 (NE) residue of domain 6 to Glu272 (OE1/OE2 ) of domain 2. In 4ENZ and 2J5W PDB-structures, the Glu272 (OE1/OE2)...His 1026 (NE) distances are 2.99 and 2.71 Å respectively. During the simulation, Glu272 interacts directly with His1026 (NE) upto ~7.22 ns thus forming a salt-bridge (Glu272...His1026(NE)) as it was observed in the both 2J5W and 4ENZ X-ray structures and the distance is ranging from 2.70 to 3.34 Å. However, after that period the oxygen atom of that acidic residue seems to be far away from that His 1026 due to variation of torsion angles of Glu272 from ~ - 66.10 (χ1), ~ 174.20 (χ2) to ~71.10 (χ1), ~ -64.50 (χ2) (though in 4ENZ crystal structure the respective torsion angles are -71.070 (χ1) and 166.010 (χ2)), however the residue Glu272 of domain 2 and His1026 of domain 6 are connected by a conserved water center W5 through hydrogen bonds (Glu272(OE1/OE2)...W5...His1026(NE)). The Glu272...W5 and W5...His1026 distances are ranging from 2.55 to 2.91 and 3.02 to 3.45Å. All these interaction and distances of water molecules from the Glu272 and His1026 residues during simulation are given in Table 2. The average energy ranges of Glu272(OE2)...His1026(NE2), Glu272(OE2)...W5 and W5...His1026(NE2) interaction are -0.107 to -0.143, -0.113 to -0.151 and -0.110 to -0.122 kcal/mol respectively. Thus recognition of T-1 copper centers belonging to D6 and D2 domains could be followed either through direct salt-bridge or water-mediated hydrogen bonding interaction between Glu272 and His1026 residues, which are depicted in (a) and (b) .

(a) CuPR-His276-Val275-Asp274-Val273-Glu272...His1026-CuCis-His (O2-bound X-ray structure)

(b) CuPR-His276-Val275-Asp274-Val273-Glu272...W5...His1026-CuCis-His (O2-bound simulated structures)

So, MD-simulation studies have indicated the interaction potentiality of two water molecule (W1 and W2) with the T1 copper center CuRS (or Cu1106) in O2-bound CP structure, which was not observed in the X-ray structures of that enzyme. Moreover one water molecule seems to be present at ~3.2 to 4.3Å away from each of the CuCis-His and CuPR centers, however, they were not been observed in the X-ray structures. Possibly due to the presence of an inadequate number of water molecules (86 number in 4ENZ and 341 number in case of 2J5W PDB structures) the interaction was missing in the crystal structure. Generally, the results have provided evidence on the involvement of some conserved/ semiconserved water molecules (W3, W4 andW5) in the inter-domain ( D6...W3...D4, D4...W4/ W4/...D2, and D2...W5...D6) recognition of type-1 copper bound His residue of one domain with Glu residues of other domain through water-mediated hydrogen bonds (Cu- His...W...Glu). The salt-bridge (Cu-His...Glu) mediated recognition of mononuclear copper centers (of 2, 4 and 6 domains) which are generally found in X-ray structures have also been observed in few snapshots during the simulation of hydrated O2-bound hCP structure. However, it is interesting to note that the salt-bridge mediated inter-domain recognition was also been observed (except D4-D2 recognition) in the 25ns simulated structure of 2J5W PDB structure which contains only 341 number of water molecules 19. Possibly, an association of water molecules in the coupling between Glu residue of one domain with Type1 Cu-bound His residue of other domain may create some hydrophilic environment in that inter-domain gap or near to the surroundings of type-1 copper centers which may be important for the stabilization of ceruloplasmin structure in the hydrated condition.

## Conclusion

MD-simulation studies of O2-bound human ceruloplasmin structure have shed some light on the role of some conserved / semi-conserved water molecules in the hydration of type-1 mononuclear copper centers and their interdomain recognition, which was not explored in the crystallographic works. The hydration potentiality of type-1 copper CuRS center is observed to be unique. Two water molecules (W1 to W2) are interacting with the copper center of remote site (CuRS). The study also reveals the interdomain recognition of three type-1 copper centers (CuPR , CuRS and CuCys-His) of D2, D4 and D6 domains, where the Cu-bound Hisresidue of one domain interacts with the Glu residue of other complimentary domain through conserved/ semi-conserved watermediated hydrogen bonding interaction (Cu-His-W-Glu). In O2- bound structure, in most of the time water molecules at the W3, W4 and W5 hydrophilic sites are playing role in interdomain recognition (D6...W3...D4, D4...W4...D2 and D2...W5...D6), and thus connected the Cu-His of one domain to Glu of other domain through hydrogen bonds. So coupling of type1 Cu-bound Hisresidue to Glu-residue of the two even-numbered complimentary domains through water-mediated (Cu-His...W...Glu) interaction in MD- simulated structure and salt-bridge (Cu-His...Glu) interaction in the X-ray structures have indicated some plausible rational on the Cu-His...W...Glu ↔ Cu-His...Glu transition during the interdomain recognition of type-1 copper centers, which might have some importance in biology and chemistry of ceruloplasmin.

## Figures and Tables

**Table 1:** Distances (�) of conserved water molecules from the mononuclear copper centers of different domains at different time (ns) during simulation of O2-bound ceruloplasmin structure. The conserved water centres which play role in the interdomain recognition of T1 copper centers are also included.

T1-copper centers (interact with water centers). Conserved water mediated Inter-domain (D) recognition.	Conserved water molecular sites	Id .No. of water molecules occupied at the conserved water molecular sites (W) and their distances from the T-1 copper centers (within bracket) at different time (ns) of MD-simulation. Id. No of water molecules (occupied at the conserved water centers) involve in inter domain recognition.					Occupation frequency (%) of the conserved or semiconserved water centers.
		3	6	9	12	15	
Cu1102 (CuPR)	W (CuPR)	W7769	W7769	W7769	W7769	W7769	~60
		-3.75	-3.22	-4.04	-3.47	-3.2	
Cu1106 (CuRS)	W1	W5605	W5605	W5605	W5605	W5605	~95
		-3.13	-2.76	-3.58	-2.92	-2.99	
	W2	W6525	W6525	W6525	W6525	W6525	~100
		-2.45	-2.53	-2.45	-2.33	-2.64	
Cu1107 (CuCys-His)	W	W8205	W8205	W8205	W8205	W8205	~60
	(CuCys-His)	-3.48	-3.9	-4.05	-4.33	-4.07	
Interdomain recognition between D6 to D4	W3	W645	W7296	-	W8333	W3460	~60
							
Interdomain recognition between D4 to D2	W4	W6968	W6736	W6736	W6859	W1927	~100
	(W4/)				(W4075)	(W8601)	
Interdomain recognition between D2 to D6	W5	-	-	W6431	W3462	W3982	~60

**Table 2:** The salt-bridge and water mediated interdomain recognition between the type-1 copper bound His- residues of one domain to Glu residue of other domain during MDsimulation of ceruloplasmin. All the hydrogen bonding distances are given in ? unit. * At 12 and 15ns snapshots, two water molecules (W4 and W4/) involve in the inter- domain recognition (His324NE2���W4���W4'���Glu633OE2).

Inter domain recognition	Interdomain recognition: Interaction between the acidic and basic residues, water mediated H- bonding interaction between His and Glu residues.	Distances (�) in the crystal. 4ENZ	2J5W	The distances of salt-bridge and water mediated H- bonding interaction of the Glu with other basic residues at different time (ns) of simulation. (The Id. No. of conserved water molecules are given in Table 1.)				
				3	6	9	12	15
Recognition of D6 of D4	Glu971OE1/OE2???His 685NE2	3.36	2.8	5.48 (OE1)	5.59 (OE1)	3.26 (OE1)	4.09 (OE2)	4.55 (OE1)
		(OE2)	(OE2)					
	Glu971OE1/OE2???W3/W3???His 685NE2	-	-	2.80 (OE1)/ 3.40	2.76 (OE1)/ 3.33	-/-	2.66 (OE2)/ 2.78	2.93 (OE2)/ 3.10
Recognition of D4 to D2	Glu633OE1???His324NE2	4.11	3.58	3.50 (OE1)	5.84 (OE1)	5.39 (OE1)	7.32 (OE1)	8.61 (OE1)
		(OE1)	(OE1)					
	Glu633OE1/OE2???W4/W4�His324NE2	-	-	2.93 (OE1)/ 3.35	2.62 (OE2)/ 3.48	2.69 (OE2)/ 3.51	-/-	-/-
	*Glu633???W4//W4/???W4/W4???His324NE2						2.69/2.82/3.73*	2.79/2.91/3.21*
Recognition of D2 to D6	Glu272OE1/OE2???His 1026NE2	2.99	2.71	2.92 (OE2)	3.21 (OE1)	4.89 (OE1)	4.56 (OE1)	5.12 (OE1)
		(OE1)	(OE2)					
	Glu272OE1/OE2???W5/W5???His1026 NE2	-		-/-	-/-	2.82 (OE1)/ 3.43	2.81 (OE2)/ 3.02	2.55 (OE2)/ 3.28
